# "Protective effects of artichoke extract and Bifidobacterium longum on male infertility in diabetic rats"

**DOI:** 10.1016/j.bbrep.2024.101834

**Published:** 2024-09-23

**Authors:** Zahra Ansari, Mohammad Hasan Maleki, Fatemeh Roohy, Zahra Ebrahimi, Mesbah Shams, Pooneh Mokaram, Zahra Zamanzadeh, Zahra Hosseinzadeh, Farhad Koohpeyma, Sanaz Dastghaib

**Affiliations:** a Department of Genetics, Faculty of Biological Sciences and Technology, Shahid Ashrafi Esfahani University, Esfahan, Iran; b Department of Biochemistry, School of Medicine, Shiraz University of Medical Sciences, Shiraz, Iran; c Department of Genetics, Islamic Azad University, Kazerun, Iran; d Department of Biology, Science and Research Branch, Islamic Azad University, Tehran, Iran; e Endocrinology and Metabolism Research Center, Shiraz University of Medical Science, P.O. Box, 71345-1744, Shiraz, Iran; f Autophagy Research Center, Department of Biochemistry, School of Medicine, Shiraz University of Medical Sciences, Shiraz, Iran

**Keywords:** Male infertility, diabetes, Anti-oxidant, Spermatogenesis, Apoptosis

## Abstract

**Background:**

Diabetes is a major global health concern and plays a significant role in male infertility and hormonal abnormalities by altering the tissue structure of spermatogenic tubes and decreasing the number of spermatogonia. This study investigated the effect of artichoke (*Cynara scolymus* L) hydroalcoholic extract and *Bifidobacterium longum* probiotic on sexual hormones, oxidative stress, apoptosis pathway, and histopathological changes in testicular tissues of diabetic rats to find an adjuvant therapy to manage the infertility complications of diabetes.

**Methods:**

In this experiment, 96 male-rats were randomly selected from eight groups. Control, Sham (normal saline), DM group (IP injected with 60 mg/kg STZ), Cynara (400 mg/kg hydroalcoholic extract of Cynara scolymus L), BBL (received 1 × 10^9^ CFU/ml/day Bifidobacterium longum), DM + Cynara, DM + BBL, and DM + Cynara + BBL groups. After 48 days of orally gavage, serum level of FBS (fasting blood sugar), Malondi-aldehyde (MDA), Total-Anti-Oxidant Capacity (TAC), FSH (Follicle-stimulating hormone), LH (Luteinizing hormone), Testosterone, Testis mRNA-expressions of *Protamin* (prm1), *BCL2*, and *Caspase-9* genes, as well as stereological changes were measured.

**Results:**

In comparison to the diabetic group, the hydroalcoholic extract of *Cynara scolymus* L combined with the probiotic *Bifidobacterium longum* resulted in a substantial decrease in FBS (p < 0.001) and MDA(p < 0.05) concentrations, and the expression of the Caspase-9 gene (1.33-fold change). In addition, serum levels of TAC, LH, FSH, Testosterone were significantly increased (p < 0.05). mRNA expression of protamine (p = 0.016) and BCL2 (0.72-fold change) were detected. Furthermore, in comparison with diabetic rats, the *Cynara scolymus L*-and *Bifidobacterium longum*-treated groups showed a significant increase in the number of sexual lineage cells, total weight, sperm count, motility, normal morphology, volume of the testis, and volume and length of seminiferous tubules (p < 0.05).

**Conclusion:**

The findings demonstrated that Cynara scolymus L extract and Bifidobacterium longum supplement had great therapeutic potential, including antioxidant, anti-apoptotic, anti-diabetic, fertility index improvement, and sex hormone modulators.

## Highlights

• Hydroalcoholic extract of Cynara scolymus L and Bifidobacterium longum improved serum antioxidant capacity in diabetic rats.
• Cynara scolymus L and probiotic Bifidobacterium *increased testis* mRNA-expressions of *Protamin* (*prm1*).
• Cynara scolymus L and probiotic Bifidobacterium *improve sexual hormones in male diabetic rats.*
• Sexual lineage cells significantly increased in diabetic rats treated with Cynara scolymus L and Bifidobacterium.

## 1 Introduction

Diabetes is one of the most common endocrine diseases caused by insufficient or resistant insulin [1]. According to epidemiological research, this disease affects 10 % of the global population, according to epidemiological research [2]. The significant global health burden posed by diabetes, given its association with severe complications such as cardiovascular disease, infertility, neuropathy, nephropathy, and retinopathy. Diabetes not only disrupts glucose metabolism but also affects various cellular processes, including autophagy, oxidative stress, AMP-activated protein kinase (AMPK) signaling, and apoptosis which are essential for cellular maintenance and survival [[3], [4], [5]].

Testicular antioxidant defense is compromised by diabetes [6,7], which leads to the accumulation of free radicals and, ultimately, apoptosis and germ cell death [8,9]. Diabetes alters the tissue structure of spermatogenic tubes and decreases the number of spermatogonia, which further adds to male infertility and hormonal abnormalities. Diabetes slows down spermatogenesis, according to several studies [10].

It has been demonstrated that diabetic rats have reduced testicular weight as well as average numbers of spermatogonia, spermatocytes, spermatids, and Sertoli cells [11,12]. Clinical and experimental research indicates that diabetes modifies the function of the pituitary-testicular hormonal axis, which lowers gonadotropin and testosterone output [13]. On the other hand, low insulin in diabetic rats may reduce the release of LH and FSH [14,15]. Moreover, insulin is necessary for the integrity of LH receptors in Leydig cells and controls cell division and metabolism in these cells. Consequently, a drop in insulin may change the activity of Leydig cells and lessen the generation of testicular steroid hormones [16].

Protamine is the main protein in sperm that attaches to DNA and is a gene involved in fertilization [17]. During sperm maturation, most histone proteins linked to DNA are replaced by prostamine. It may therefore be thought of as a molecular indicator of fertility [18]. Human sperm contains just 15 % of the testicular histones; the remaining 85 % are replaced by protamine [19]. Therefore, during reproductive processes, epigenetic modifications may have a significant effect on sperm production. Diabetes has been linked to an increased risk of oxidative stress, increased lipid peroxidation [20], oxidative DNA damage [21] and decreased antioxidant enzyme activity [22,23].

ROS have a crucial role as second messengers in spermatozoa during critical cellular processes associated with fertilization, including as sperm-oocyte fusion, acrosome response, hyperactivation, and capacitation. Therefore, sufficient male reproductive potential requires a physiological level of ROS. It is commonly known that elevated ROS levels result in peroxidative damage to spermatozoa's lipids, proteins, and DNA, which negatively impacts the sperm's ability to remain structurally and functionally intact. There are multiple lines of data that demonstrate the link between varicocele and high seminal ROS levels. It has been demonstrated that spermatozoa exposed to ROS experience oxidative damage and sperm cell death [24,25]. In contrast, oxidative stress suppresses the formation of steroid hormones and negatively impacts steroid hormone production [26,27].

Previous studies have shown that when oxidative stress arises, the release of cytochrome *c* as a result of a ruptured mitochondrial membrane triggers apoptosis. Cytochrome *c* release is controlled by the BCL-2 family of proteins, which are found in the inner mitochondrial membrane [28]. The imbalance between anti-apoptotic and pro-apoptotic groups of the BCL-2 protein family leads to cell damage resulting from the release of cytochrome *c* and the development of an apoptosome containing cytochrome *c*, Apaf-1, and caspase-9 [29].

The two main and most successful therapies for diabetes mellitus are insulin injections and hypoglycemic drugs; however, these drugs also come with a number of unfavorable side effects, including increased fat deposits, hypoglycemia shock, and atrophy of adipose tissue at the injection site. The long-term ineffectiveness of these drugs due to adverse effects slows the advancement of debilitating consequences associated with diabetes. Herbal remedies have a long history of use and are the most readily available source when it comes to treating metabolic diseases like diabetes. They also have less negative consequences. Medicinal plant extracts have antioxidant properties and can help diabetics manage the consequences of oxidative stress because they include high quantities of phenolic and flavonoid components [30,31].

Numerous published studies have tested the antidiabetic potential of various herbs with a history of use in traditional medicine. In this sense, *Cynara scolymus* L is an herb that helps diabetic patients' blood glucose levels. *Cynara scolymus* L *(Asteraceae),* known as artichokes, is a perennial species of thistle that is grown for food and is native to the Mediterranean region. It is widely used as a vegetable, and its leaves are regularly used to treat hepatitis, hyperlipidemia, obesity, and dyspeptic disorders in traditional medicine [32]. This plant can also reduce total serum cholesterol and triglyceride levels while reducing the production of reactive oxygen species (ROS), lipid peroxidation, and oxidation of low-density lipoproteins, according to in vitro investigations with patients with hypercholesterolemia [33,34]. The active ingredients in this plant are organic acids and derivatives of caffeic acid, including cyanine, chlorogenic, neochlorogenic, and cryptochlorogenic acid. The main mechanism for *Cynara scolymus* (artichoke)'s antidiabetic properties is the presence of bioactive chemicals such chlorogenic acid, according to the recent published systematic review and meta-analysis study. By enhancing insulin sensitivity and blocking glucose 6-phosphatase, an enzyme essential to the hepatic synthesis of glucose, these substances lower fasting blood sugar. Furthermore, another important mechanism that is considered is the regulation of the digestion of carbohydrates by inhibiting alpha-glucosidase [35]. On the other hand, the mentioned mechanism that *Cynara scolymus*, alleviate oxidative stress and safeguard pancreatic β-cells to increase insulin release, is by their strong antioxidant activity. Additionally, it addresses dyslipidemia—a condition frequently linked to diabetes—by lowering LDL, triglycerides, and total cholesterol while raising HDL levels. The extract also decreases hepatic glucose synthesis and increases insulin sensitivity to minimize hyperglycemia, and its anti-inflammatory qualities assist lower markers of inflammation like IL-6 and TNF-α [36,37].

Probiotics are beneficial for both treating and preventing diabetes because they come from the body's microbiome [38]. Probiotics are thought of as super-beneficial foods since they are helpful microorganisms that exert positive and healing effects on their host by establishing a microbial balance in the intestine and other body organs [39]. Probiotics have been shown to be effective in treating metabolic disorders like diabetes in various trials to date. Owing to its numerous benefits, including improving protein digestion, boosting the immune system, and preventing or reducing illnesses such as intestinal infections and diarrhea, *Bifidobacterium longum* is receiving a lot of interest in this respect [40]. Based on the recent systematic review and meta-Analysis study, there are multiple interrelated processes by which *Bifidobacterium longum* carries out its antidiabetic actions. By restoring a healthy balance to the gut microbiota, it enhances the function of the gut barrier and lowers pro-inflammatory cytokines like TNF-α and IL-6, which in turn reduces systemic inflammation. This decrease in inflammation improves hepatic glucose metabolism by lowering hepatic glucose synthesis and stimulating incretin secretion. It also increases insulin sensitivity, especially in peripheral tissues. Bifidobacterium longum also lowers oxidative stress, which shields pancreatic β-cells, and controls lipid metabolism, which helps control dyslipidemia—a condition frequently linked to diabetes. It improves blood glucose management by promoting insulin sensitivity and overall glucose regulation by reducing endotoxemia [41]. In 2022, researchers found that administering *Bifidobacterium longum* supplemented with zinc promoted reproductive systems in rats [42]. In diabetic patients, probiotics appear to be able to lower blood glucose levels, act as an adjuvant therapy to reduce insulin injection complications, increase treatment costs, and eliminate prescription adverse effects [43].

The objective of this work is to examine the impact of *Cynara scolymus* L extract and *Bifidobacterium longum* probiotic on sexual hormone (FSH, LH, and Testasterone) concentrations, oxidative stress levels (MDA, and TAC), gene expression of (*Protamin, BCL2, and Caspase-9*) and testicular histology in rats with diabetes. Through the assessment of their influence on these factors, the research aims to determine efficacious supplementary treatments for controlling reproductive problems linked to diabetes.

## 2 Methods

### 2.1 Preparation of *Cynara scolymus* L hydroalcoholic extract

*Cynara Scolymus* L (Herbarium number 5167), which was extracted in Natural Resources Department of the Hamedan Province Agricultural and Natural Resources Research Center. To obtain the hydroalcoholic *Cynara scolymus* L extract, 100 g of dry leaf powder was placed into the perqualator and left for 72 h in 500 cc of 70 % ethanol for soaking. Then, the solution was obtained from the percolator, which contained a hydroalcoholic solvent and total extractive material. A rotary device was used to separate the excess solvent (ethanol) until it became completely concentrated. Then, a desiccator and vacuum pump were employed to convert the extract into a brown powder, which was stored at 4 °C until use. The extraction efficiency was 18.28/100 g. Then (desired dose/kg) of *Cynara scolymus* was dissolved in normal saline and administered by gavage [44].

### 2.2 Preparation of *Bifidobacterium longum* (B. longum) probiotic

Lyophilized cultures of *Bifidobacterium longum* (B. *longum*) UABI-14™ was provided by Christian Hansen (Denmark). Briefly, the lyophilized powder was grown to a density of 2–4 x10^9^ CFU/mL and centrifuged to generate cells to manufacture the B. longum probiotic solution. The collected cells (1 × 10^9^ CFU/mL) were suspended in normal saline [45,46].

### 2.3 Animals

Eight-week-old male Sprague-Dawley adult rats weighing 150–200 g were acquired from Shiraz University of Medical Sciences' animal laboratory. They were housed in standard settings, with a 12-h light/dark cycle, 25–35 % humidity, and 20–22 °C temperature. The Shiraz University of Medical Sciences Ethnic Committee approved all procedures in accordance with the NIH standard (animals care and use protocol; NIH publication No. 85-23, revised in 1996).

### 2.4 Diabetes induction

Streptozotocin (STZ) was administered intraperitoneally (IP) at a dose of 60 mg/kg to induce diabetes in rats [47,48]. To induce diabetes (prepared 5 min prior to injection), STZ was dissolved (freshly, light-protected) in 0.9 % sodium chloride and 100 mM sodium citrate buffer at pH4. Fasting blood glucose levels were measured 7 days after STZ injection in the lateral rat tail vein and examined using a glucometer (Accu_chek) to confirm the induction of type 1 diabetes. Diabetic rats with blood sugar levels greater than 300 mg/dl were used in this study [49].

### 2.5 Experimental design

Based on the relevant published study, the minimal sample size needed was calculated, taking into account the 20 % dropout rate of 12 rats in each group [50].

96-male rats were divided equally into eight groups for this investigation.

- Control group: The animals were treated with water and normal-diet only -Sham group: Animals received only the compound solvent (normal saline) by gavage.
- Diabetic control group (DM): The rats were injected with STZ and received 1 ml of normal saline by gavage. Experimental group 1 (Cynara): Healthy rats received 1 ml of 400 mg/kg hydroalcoholic extract of *Cynara scolymus* L by gavage [44].
- Experimental group 2 (BBL): In this group, healthy rats received 1 ml of *Bifidobacterium longum* by gavage (1 × 10^9^ CFU/ml/day) [51].
- Experimental group 3 (DM + Cynara): Diabetic rats received 1 ml of 400 mg/kg hydroalcoholic extract of *Cynara scolymus* L by gavage.
- Experimental group 4 (DM + BBL): In this group, diabetic rats received 1 ml of *Bifidobacterium longum* by gavage (1 × 10^9^ CFU/ml/day) by gavage.
- Experimental group 5 (DM + Cynara + BBL): In this group, diabetic rats received 0.5 ml of 400 mg/kg hydro alcoholic extract of *Cynara scolymus* L and 0.5 ml of *Bifidobacterium longum* by gavage (1 × 10^9^ CFU/ml/day) by gavage.

**Fig. 1:**
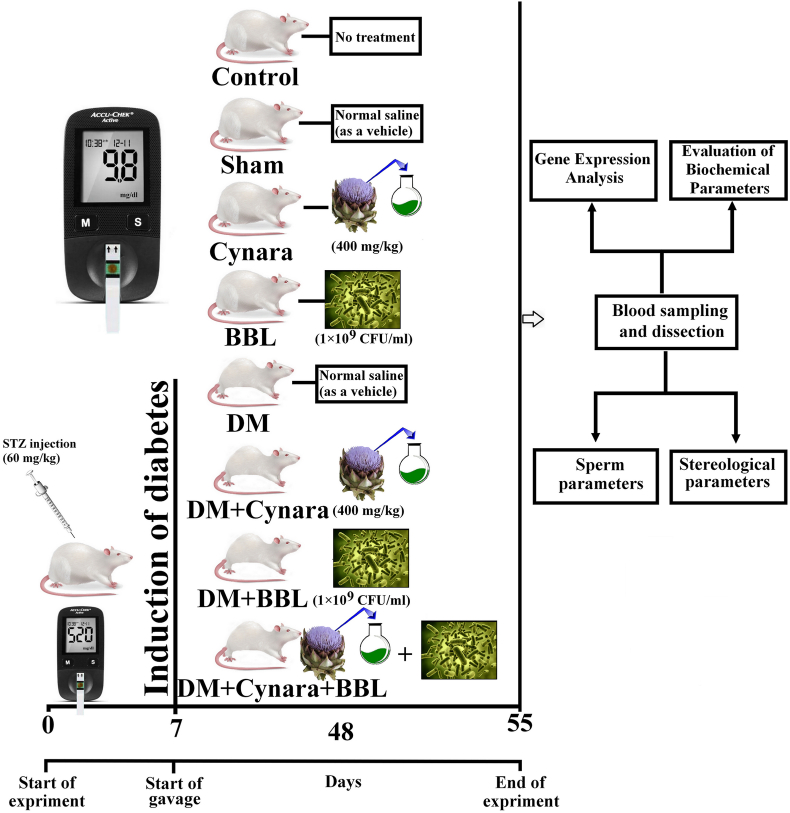
Schematic representation of experimental protocol.

A schematic figure of experimental protocol is illustrated in Fig. 1.

It should be noted that the rats underwent treatment seven days following their STZ injection. Following the 48-day treatment period [52,53], the animals were put to sleep with a mixture of ketamine (80 mg/kg) and xylazine (5 mg/kg). Using a heart puncture, 5 mL of blood were extracted. Samples of blood were gathered and placed in centrifuge tubes. After coagulating for 20 min at room temperature and centrifuging for 15 min at 3000 rpm, their serum was separated. The left testis and epididymis were removed for sperm and stereological investigation, respectively, following blood work. Analysis was done on gene expression. After removal, the right testis was kept frozen at −80 °C. As per the directives of Shiraz University of Medical Sciences' ethical committee, every animal was put to death by being enclosed in a carbon dioxide chamber.

### 2.6 Evaluation of biochemical parameters

Serum fasting blood sugar (FBS) (mg/dl) levels were quantified by the calorimetric method using kits supplied by Biosystems, Spain, using a prestige instrument (Hitachi, Japan). Serum follicle-stimulating hormone (FSH) (mIU/ml), luteinizing hormone (LH) (mIU/ml), and testosterone (ng/ml) levels were assessed using an ELISA kit for rats (Bioassay Technology Laboratory, China) [49].

### 2.7 Measurement of total-antioxidant-capacity (TAC) and malondialdehyde (MDA)

Total serum antioxidant levels were measured using a spectrophotometric method at controlled temperature (Kia-Zist, Iran). In this method, 2-2-azino-di3-ethyl benzothiazoline sulfonate (ABTS) was incubated with peroxidase and H2O2 to produce ABTS cation radicals, which have optical absorption at 600 nm. Antioxidants in the sample inhibit color production, and the concentration is reported in millimoles per liter. MDA was measured using the calorimetric method (Kia-Zist, Iran). In this reaction, the lipid peroxidation product (MDA) creates a colored complex with the TBA reagent, which has the maximum amount of light absorption at 532 nm [54].

### 2.8 Examination of sperm parameters (sperm count, motility, viability morphology)

To check the motility and sperm count, 1 cm from the left cauda epididymis was extracted and kept in 5 ml of Hanks solution to create a suitable environment for the survival of sperm in a short period of time.

Sperm were divided into three categories according to the type of movement.

- Progressive movement
- Non progressive movement
- Immotile

To count sperm numbers, one drop of the sperm-containing solution was placed on the neobar lam. The number of sperms located on the four big squares was counted to determine the average number of sperms. The total number of extricated sperms from 1 cm of the cauda epididymis was calculated according to the following formula:

A = B.C.D

A: The total number of extricated sperms from the cauda epididymis
B: The number of counted sperms in 0.1 mm3 of the solution.
C: Depth factor = 10
D: Dilution factor = 5000 mm3 (sperm present in 1 cm of cauda epididymis are released in 5 ml of solution) [54].

### 2.9 Evaluation of sperm viability

The viability of the sperm was evaluated by eosin-nigrosin staining. Eosin and nigrosin (both produced by Merck, Darmstadt, Germany) were made with distilled water. Two volumes of 1 % eosin were combined with one volume of sperm suspension. After 30 s, an equivalent volume of nigrosin was added to the mixture. Thin smears were then made and examined at a 40 × magnification using a light microscope (Nikon E−200, Japan). In this way, non-viable sperm colored red while viable sperm remained white [54].

### 2.10 Sperm morphology analysis & estimated percentage of abnormal sperms

The suspension was placed on a slide, stained for 5–10 min with 1 % eosin Y, and allowed to dry. In all, 100–200 spermatozoa per rat were counted in each sample, and the percentage of abnormal sperm was calculated. Amorphous heads, two heads, merged bodies, two tails, and a normally formed head with a damaged or twisted tail were considered abnormal sperm [55,56].

## 3 Gene expression analysis

The mRNA expression levels of *prm1* (protamine), *Bcl-2* and *Caspase-9* were evaluated using quantitative RT-PCR with an ABI 7500 real-time PCR system (USA) and SYBR Green Master Mix (Amplicon, Denmark). Total RNA was extracted using (KiyanZOLTotal RNA Extraction) Kit (Kiyandanesh Company, Shiraz, Iran). cDNA was synthesized from 1 μg of total RNA using the Sinacolon kit (Sinacolon, Tehran, Iran). The CT values of *Bcl-2* and *Caspase-9* genes were normalized to GAPDH (glyceraldehyde-3-phosphate dehydrogenase) and prm1 (protamine) normalized with internal control B2m (Beta-2-Microglobulin) as an internal control gene for obtaining relative expression level using comparative Ct (2^−ΔΔCt^) method [57]. All samples were evaluated in triplicate and ddH2O served as a no-template control (NTC). The primer pair sequences of GAPDHI and the mentioned genes were designed using AlleleID 7.73 software respectively as follows:.

**Table 1:** The primers sequences and PCR product lenght Table 1. The primers sequences and PCR product lenght.

Primer	Sequences (5'->3′)	PCR Product length
*Cas9: F*	ACATCTTCAATGGGACCGGC	85bp
*Cas9: R*	TCTTTCTGCTCACCACCACAG	
*GAPDH: F*	AAAGAGATGCTGAACGGGCA	100bp
*GAPDH: R*	ACAAGGGAAACTTGTCCACGA	
*Bcl-2: F*	GGAGGATTGTGGCCTTCTTT	100bp
*Bcl-2: R*	GTCATCCACAGAGCGATGTT	
*Prm1: F*	ATGGCCAGATACCGATGCTGC	80 bp
*Prm1: R*	CTCCTCCGTCTGCGACATCTTC	
*B2m: F*	CGTGCTTGCCATTCAGAAA	244 bp
*B2m: R*	ATATACATCGGTCTCGGTGG	

### 3.1 Stereological parameters

Six rats from each group were randomly selected for stereo-logical examination of their testes stereo logically examined. The left testis was removed. The primary volume was determined using Scherle's method by submersion in distilled water. It was subsequently fixed in formaldehyde solution buffer. An orientator approach was used to prepare these portions. Every testis yielded 8–12 pieces. The area of any testicular portion was determined after a circle was punched out of the testis with a trocar. Hematoxylin and eosin was used to stain slabs and circular sections that had been processed, sectioned (4 and 25 m thick), and stained [58]. The area of the circular piece and shrinkage factor "d(sh)" were then recalculated.

Degree of shrinkage = 1-(AA/AB)^1.5^

AA and AB indicate the areas of the circular pieces after processing and staining, respectively. The sections were examined under a video microscope (Nikon E200, Japan). A systematic random sampling method was used to collect samples from microscopic fields. Then, university-developed stereology software, a point grid, and an unbiased dissector frame were superimposed on a monitor with a microscopic image.

### 3.2 The volume estimation of the testis component

Using 5 m sections, the point counting method was used to calculate the Vv (structural/testicular) volume density of the seminiferous tubules and interstitial tissue. The total number of points touching the seminiferous tubules, epithelium, interstitial tissues, and lumen was added together. The sum of those points were divided by the total number of points hitting the entire testis [59]. Each structure's overall volume was calculated according following formula:

$$Vv\left(structure\right)=\sum_{i=1}^{n}p\frac{\left(structure\right)}{\sum_{i=1}^{n}\left(reference\right)}$$

Where the $\prime \prime {\sum_{i=1}^{n}p\;\left(structure\right)}^{\prime \prime }$ was number of points hitting interstitial tissue profiles.The seminiferous tubule and $\prime \prime {\sum_{i=1}^{n}\;\left(reference\right)}^{\prime \prime }$ was the number of total points hitting testis sects. The total intended structural volume obtained by multiplying the density in final trabecular volume:

V(_structure_) = Vv_(structure/testis)_ × V_final_

### 3.3 Assessment of number of different cells of testis

Using "optical dissection" technique on 25 m thick sections, the numerical density Nv(cells/testes) and total number of spermatogonia, spermatocytes, round and long spermatids, Sertoli and Leydig cells were determined. The dissector method was utilized to calculate "Nv(cells/testicle)" using the stereology program Microcator (Heidenhain MT-12, Leipzig, Germany) and a high-numerical-aperture oil-immersion objective lens. A z-axis distribution plot was created to determine the guard zone and height of the dissector in tissue sections after recording the distribution of all cells extracted at various focal planes [56]. The following formula was used to calculate numerical density, the number of cells per unit volume in germinal epithelium, abbreviated as "Nv(cells/testicles)":

$$Nv=\frac{\sum_{i=1}^{n}Q}{\sum_{i=1}^{n}P\times h\times \left(\frac{a}{f}\right)}\times \frac{t}{BA}$$

ΣQ is the number of nuclei under investigation, ΣP is the total area of unbiased counting frame in all fields, h is the dissector height, t is the average section thickness, and BA: shows the microtome setting. Using following formula, total number of cells was estimated.

N(cell) = Nv(cell/testis) × V(epithelium)

### 3.4 Tubular length assessment

Here is how tubule length density (Lv) was determined:

$$Lv=\frac{2\sum_{i=1}^{n}Q}{\sum_{i=1}^{n}P\times \left(a/f\right)}$$

“ΣQ” represents total number of counted tubule profiles per animal testicle, “ΣP” represents total number of counted frames in each rat. “a/f” suggests the area of the counting frame. The following formula calculated total length of tubules “L(tubules)":

L(tubules) = LV (tubules/testicle) × V(testis)

### 3.5 Statistical analysis

The data were analyzed by the SPSS software (version 20, IBM Inc., USA). Normality of the data was assessed using the Shapiro-Wilk test. Based on the normality of the data for statistical analysis, one-way analysis of variance (ANOVA) along with Tukey's post-hoc test was utilized. A P-value of less than 0.05 was deemed statistically significant. The results were reported as mean ± Standard Deviation (SD).

## 4 Results

### 4.1 -Determination of serum biochemical parameters FBS, FSH, LH, and testosterone

**Fig. 2:**
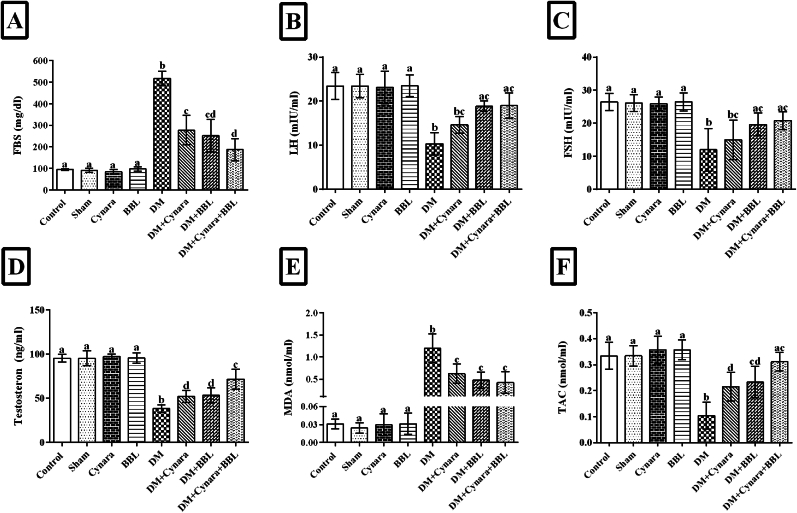
Assessment of the serum biochemical variables (A) FBS, (B) LH, (C) FSH, (D) testosterone, (E) MDA, and (F) TAC in the experimental groups after 48 days. Control, Sham, DM group (IP injected with 60 mg/kg STZ), Cynara group (healthy group receiving 400 mg/kg hydro-alcoholic extract of *Cynara scolymus* L), BBL group (healthy group receiving 1 × 10^9^ CFU/ml/day *Bifidobacterium longum*), DM + Cynara group (diabetic group receiving hydro-alcoholic extract of *Cynara scolymus* L), DM + BBL group (diabetic group receiving hydro-alcoholic extract of *Cynara scolymus* L and *Bifidobacterium longum*). Data are presented as mean ± SD, n = 12. ^a,b,c,d.^ There was no significant difference between columns, which have at least one similar letter. However, dissimilar letters indicate a significant difference (p < 0.05).

Compared to the other groups, the diabetic group had a significantly higher mean FBS concentration (P < 0.001). The FBS levels were significantly lower in the diabetic rats (P < 0.001) compared to the healthy rats when they were given Cynara extract, BBL, or a combination of Cynara and BBL. This is shown in Fig. 2A. The results displayed in Fig. 2B–D indicate that the diabetic group had significantly lower serum levels of LH, FSH, and testosterone (P < 0.05) than the other healthy groups (control, sham, Cynara, and BBL). The DM + BBL, DM + Cynara, and DM + Cynara + BBL groups had significantly greater levels of LH, FSH, and testosterone than the diabetic group (P < 0.05).

### 4.2 -Hydroalcoholic extract of Cynara scolymus L with probiotic Bifidobacterium longum improved serum antioxidant capacity in diabetic rats

Our findings show that diabetes dramatically reduced TAC levels while also increasing serum MDA levels in comparison to other groups (P < 0.001) (Fig. 2E and F). TAC levels significantly increased and MDA levels dropped in the DM + BBL, DM + Cynara, and DM + Cynara + BBL groups relative to the diabetic group (p < 0.05). The combination of Cynara extract and BBL enhanced and reversed the effects of STZ on these parameters, which is consistent with the study's findings.

### 4.3 -Effects of hydroalcoholic extract of Cynara scolymus L with probiotic Bifidobacterium longum on mRNA expression levels in *BCL-2*, *Caspase-9* and *protamin* (*prm1*) genes

**Fig. 3:**
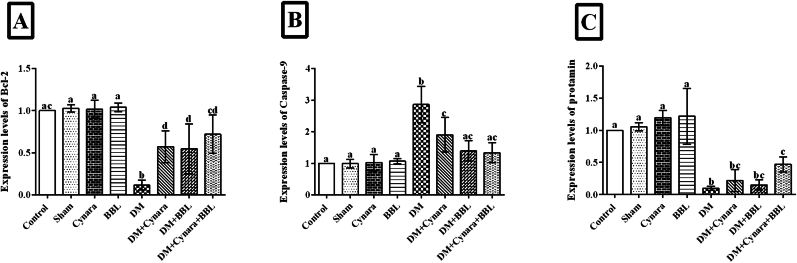
After 48 days of therapy, the mRNA expressions of (A) *BCL2*, (B) *Caspase-9*, and (C) *Protamine* in several experimental groups of rats are shown. Real-time PCR was used to assess the mRNA expressions in the Control, Sham, DM group (IP injected with 60 mg/kg STZ), Cynara (healthy group receiving 400 mg/kg hydro-alcoholic extract of *Cynara scolymus* L), BBL (healthy group receiving 1 × 10^9^ CFU/ml/day *Bifidobacterium longum*), DM + Cynara (diabetic group receiving hydro-alcoholic extract of *Cynara scolymus* L (diabetic group receiving hydro-alcoholic extract of *Cynara scolymus* L and *Bifidobacterium longum*). Data have been presented as mean ± SD. ^a,b,c,d^.There were no significant differences between the columns containing at least one similar letter. However, different letters reveal a significant difference (p < 0.05) **.**

BCL-2 expression levels were considerably lower than those in the healthy groups, as shown in Fig. 3A (p < 0.001). A noteworthy rise was noted in the treated diabetic groups (DM + Cynara DM + BBL and DM + Cynara + BBL) relative to the diabetic group (0.57-, 0.54-, and 0.72-fold changes, respectively). Fig. 3B illustrates that the diabetes group had a significant increase in Caspase-9 mRNA expression (p < 0.001) when compared to the control, sham, Cynara, and BBL groups. In contrast to the diabetic group, the DM + Cynara, DM + BBL, and DM + Cynara + BBL groups displayed significantly lower Caspase-9 mRNA expressions (1.90-, 1.40-, and 1.33-fold changes, respectively) (p < 0.001). Compared to other healthy groups, the diabetes group had reduced protamine (prm1) gene expression. On the other hand, protamine mRNA levels increased significantly (p = 0.016) in the DM + Cynara + BBL group but were not as high as in the control and sham groups (Fig. 3C).

### 4.4 -Hydroalcoholic extract of Cynara scolymus L with probiotic Bifidobacterium longum improved sperm parameters

**Fig. 4:**
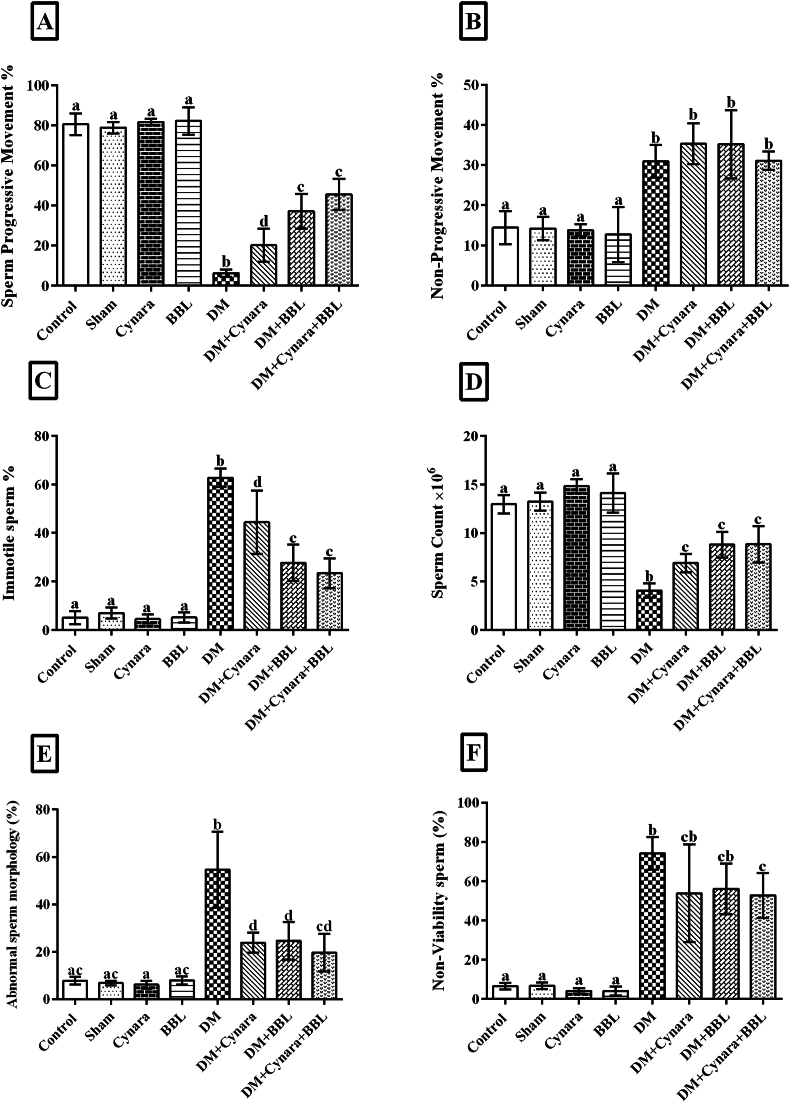
Evaluation of sperm parameters in various experimental groups, including the Control, Sham, DM group (IP injected with 60 mg/kg STZ), Cynara (healthy group receiving 400 mg/kg hydro-alcoholic extract of *Cynara scolymus* L), BBL (healthy group receiving 1 × 10^9^ CFU/ml/day *Bifidobacterium longum*), DM + Cynara (diabetic group receiving hydro-alcoholic extract of *Cynara scolymus* L (diabetic group receiving hydro-alcoholic extract of *Cynara scolymus* L and *Bifidobacterium longum*) after 48 days of treatment. (A) percentage of sperm progressive movement, (B) percentage of sperm non-progressive movement, (C) percentage of immotile sperm, (D) sperm count, (E) percentage of abnormal sperm morphology, and (F) percentage of non-viability sperm. Data are presented as mean ± SD, n = 12. ^a,b,c,d^ There were no significant differences between the columns containing at least one similar letter. However, different letters reveal a significant difference (p < 0.05).

According to Fig. 4A–F The sperm parameters of the control, sham, Cynara, and BBL groups did not differ from those of the other healthy groups. The percentage of immotile sperm and aberrant sperm morphology increased significantly (p < 0.001) in the diabetes groups when compared to the other healthy groups, according to the data. Nevertheless, these characteristics decreased after diabetic rats were treated. Sperm count and percentage of progressive sperm movement were significantly lower in the diabetic groups compared to the healthy groups (p < 0.001). The sperm count and the percentage of progressive sperm movement were significantly higher in the diabetic groups treated with Cynara, BBL, and Cynara + BBL (p < 0.01) than in the healthy rats. A higher number of diabetic rats (p < 0.001) was observed in the results when compared to the other healthy groups. However, the percentage of non-progressive movement improved and did not revert in the diabetic groups (DM + Cynara, DM + BBL, and DM + Cynara + BBL).

The DM + Cynara, DM + BBL, and DM + Cynara + BBL groups with the diabetic one did not differ from one another. On the other hand, the percentage of non-viable sperm in diabetic rats was much lower than in healthy rats (p < 0.001), but it increased dramatically in diabetic rats treated with a combination of Cynara and BBL.

### 4.5 -Stereological study

#### 4.5.1 -Effects of *Cynara scolymus* hydroalcoholic extract and probiotic *Bifidobacterium longum* on testis volume, seminiferous tubule volume, germinal epithelium volume, interstitial tissue volume and seminiferous tubule length

**Fig. 5:**
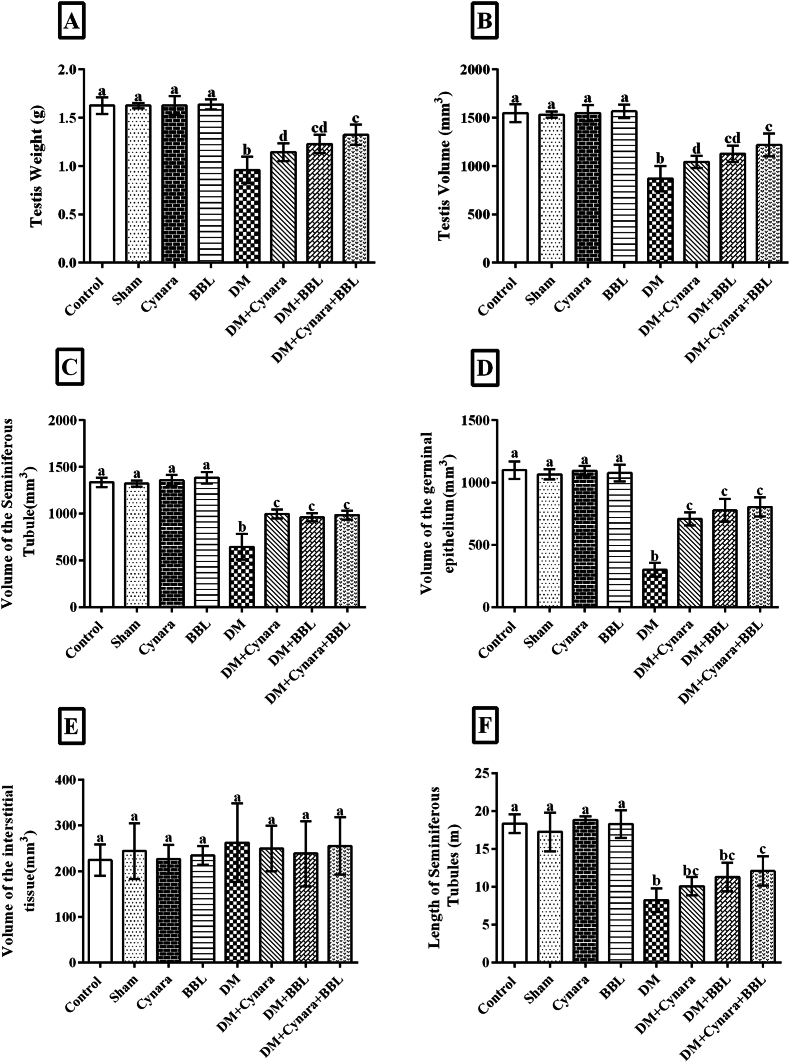
Evaluation of the (A) testis weight(g), (B) testis volume (mm^3^), (C) volume of the seminiferous tubules(mm^3^), (D) volume of germinal epithelium(mm^3^), (E) volume of interstitial tissues(mm^3^), and (F) length of the seminiferous tubules (m) in different experimental groups, including the Control, Sham, DM group (IP injected with 60 mg/kg STZ), Cynara (healthy group receiving 400 mg/kg hydro alcoholic extract of *Cynara scolymus* L), BBL (healthy group receiving 1 × 10^9^ CFU/ml/day *Bifidobacterium longum*), DM + Cynara (diabetic group receiving hydro-alcoholic extract of *Cynara scolymus* L (diabetic group receiving hydro-alcoholic extract of *Cynara scolymus* L and *Bifidobacterium longum*) after 48 days of treatment. Data are presented as mean ± SD, n = 12. ^a,b,c,d^.There were no significant differences between the columns containing at least one similar letter. However, different letters reveal a significant difference (p < 0.05)**.**

**Fig. 6:**
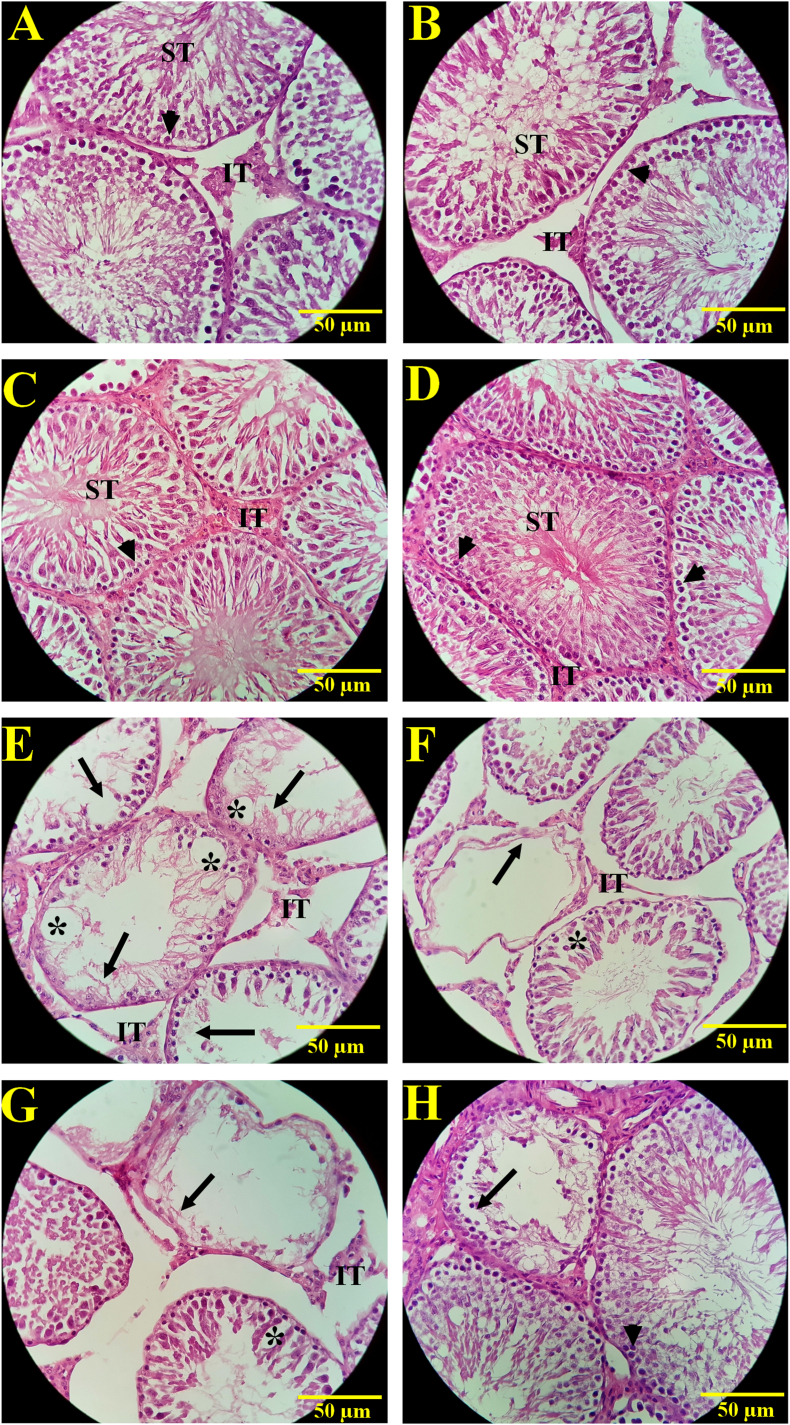
A photomicrograph of histopathological alterations in the testicular tissue in each of the study groups: Control group (A), sham group (B), healthy group receiving hydro-alcoholic extract of *Cynara scolymus* L (C), healthy group receiving *Bifidiobacterium longum* (D), diabetic group (E), diabetic group receiving hydro-alcoholic extract of *Cynara scolymus* L (F), Diabetic group receiving *Bifidiobacterium longum* (G), diabetic group receiving *Cynara scolymus* L + *Bifidiobacterium longum* (H). The germ cells (arrowhead) and tubules (ST) in the control, sham, and healthy groups treated with Cynara and BBL are not showing any pathological damage in the image above. So that the spermatogonial cells, spermatocytes, and long/round spermatids fill the tubules (ST) in a regular manner he testicular tissue of the diabetic group (E), however, shows signs of atrophy and shrinking of the tubules, destruction of the germinal epithelium (arrow sign), a considerable decrease in number of germ cells, and presence of empty areas inside the tubules (star sign). A considerable rise in number of germinal cells was seen in the diabetic groups treated with hydro-alcoholic extract of *Cynara scolymus* L and the probiotic *Bifidiobacterium longum*, but it was not identical to control group On the other hand, some areas of the testicular tissue still exhibited presence of tubules empty of cells and seminiferous tubule shrinkage (arrow sign). (IT also shows the interstitial tissue that is edematous in some parts in diabetes groups yet in the diabetic group receiving *Cynara scolymus* L + *Bifidiobacterium longum* edema of interstitial tissue was improved). H&E staining, magnification 400×.

Fig. 5, Fig. 6 show that the weight and total volume of the testes, seminiferous tubules, germinal epithelium, and interstitial tissue significantly decreased in the diabetic group (p < 0.001) compared to the control, sham, Cynara, and BBL groups.

Nonetheless, in comparison to the diabetic group, these variables were considerably higher in the DM + Cynara, DM + BBL, and DM + Cynara + BBL groups (p < 0.05). Interestingly, the interstitial tissue volume did not significantly change between the treated groups.

#### 4.5.2 -Effects of hydroalcoholic extract of Cynara scolymus L with probiotic Bifidobacterium longum on number of sexual lineage cells

**Fig. 7:**
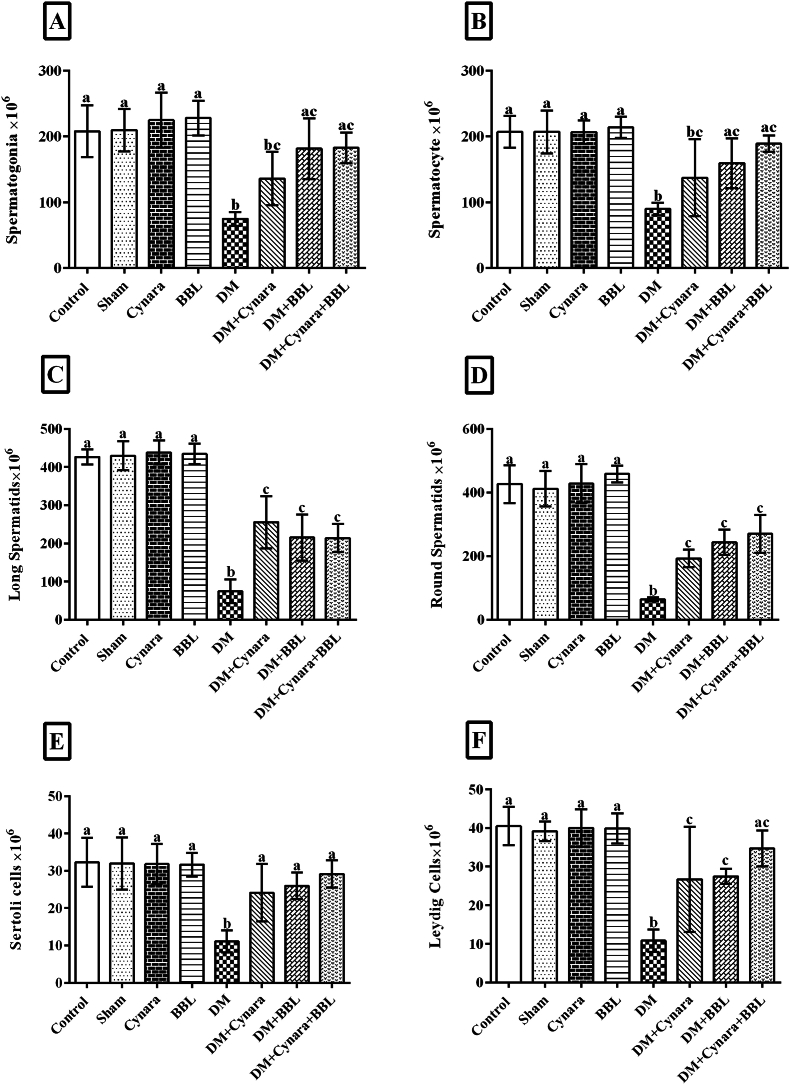
Evaluation of the number of A) spermatogonia, B) spermatocytes, C) long spermatids, D) round spermatids, E) Sertoli cells, and F) Leydig cells in different experimental groups: Control, Sham, DM group (IP injected with 60 mg/kg STZ), Cynara (healthy group receiving 400 mg/kg hydro-alcoholic extract of *Cynara scolymus* L), BBL (healthy group receiving 1 × 10^9^ CFU/ml/day *Bifidobacterium longum*), DM + Cynara (diabetic group receiving hydro-alcoholic extract of *Cynara scolymus* L (diabetic group receiving hydro-alcoholic extract of *Cynara scolymus* L and *Bifidobacterium longum*) after 48 days of treatment. ^a,b,c,d.^ There were no significant differences between the columns containing at least one similar letter. However, different letters reveal a significant difference (p < 0.05).

In comparison to the control, sham, Cynara, and BBL groups, the diabetic group exhibited significantly fewer spermatogonia (p < 0.001), spermatocytes (p < 0.001), long and round spermatids (p < 0.001), Leydig cells (p < 0.001), and Sertoli cells (p < 0.001) (Fig. 6, Fig. 7F). In diabetic rats given Cynara, BBL, or Cynara + BBL, all sexual lineage cells expanded significantly. Notably, there was no discernible difference in any of these parameters between the Cynara, BBL, sham, and control groups.

## 5 Discussion

The most significant finding of this study is that the administration of *Cynara scolymus* L plant extract and *Bifidobacterium longum* could optimally reduce diabetes-related complications leading to infertility in diabetic male rats. With the combination of these components, we observed an even more effective therapeutic potential for diabetes-related infertility, as evidenced by the following factors: lipid peroxidation inhibition, increased total antioxidant serum levels, improved sex hormones (FSH and LH), sperm movement, stereological findings, modulated levels of *protamine (prm1)* expression, and apoptosis-related genes *(caspase 9, BCL-2).* Previous research has revealed that oxidative stress in diabetes damages testicular tissues in diabetic rats [60].

The primary goal of the study is to determine how Bifidobacterium longum impacts the breakdown and release of Cynara scolymus L's beneficial metabolite including phenolic and flavonoid compounds that's helpful in the treatment of diabetes. High ROS levels have been linked to infertility in up to 40 % of men. Furthermore, additional research in this area has found that high levels of ROS are linked to infertility in 30–80 % of men. Oxidative stress (OS) damages the reproductive system and sperm, resulting in decreased sperm motility, lipid peroxidation, and oocyte-sperm fusion, as well as increased DNA damage. Several studies have found that ROS has a significant impact on spermatogenesis and sperm function. They discovered that excessive ROS generation harmed sperm motility, morphology, and concentration, as well as caused DNA damage and apoptosis [61,62].

Flavonoids have been shown to reduce the number of free radicals and, hence, the complications caused by diabetes. Miraj and colleagues discovered that the *Cynara scolymus* L plant possesses antioxidant and anti-diabetic properties, and that the compounds contained in its leaves, such as cynarin and luteolin, play an important role in lowering blood glucose and cholesterol [63]. The Protective effects of *Cynara scolymus* L plant against oxidative stress was also indicated by Downar Zapolska et al. research [64]. It has been reported that *Cynara scolymus* L has anti-hyperglycemic properties, which are mediated at least in part by antioxidant and hypolipidemic effects [65]. In type 2 diabetic rats with nonalcoholic fatty liver disease, extracts of *Cynara scolymus* L and *Silybum marianum* enhanced the glucose tolerance test and increased serum superoxide dismutase activity [33].

According to Mahmoud Ben Othman et al., obese diabetic mice treated with *Bifidobacterium longum* showed a significant decrease in body weight gain, fatty tissue mass, and blood glucose levels. In addition, a significant decrease in blood glucose levels was observed during an oral glucose tolerance test (OGTT). The medication also lowered the cholesterol, triglyceride, and non-esterified fatty acid levels. Serum and urine analyses revealed that *Bifidobacterium longum*-treated animals had reduced creatinine levels, implying that Bifidobacterium longum may have the capacity to prevent obesity and diabetes [66]. It has been proven that either type 1 or type 2 diabetes can have a negative impact on male fertility and sperm quality, including sperm motility, sperm DNA integrity, and seminal plasma components [67].

By administering *Cynara scolymus* L plant extract and *Bifidobacterium longum* separately and together in diabetes model rats, we discovered that they have antioxidant and lipid peroxidation inhibitory activities. Therefore, their favorable effects on reproductive indicators may be attributable to their antidiabetic, antioxidative, and hypolipidemic properties. Numerous studies have shown the beneficial effects of probiotics and plant extracts on spermatogenesis. Spermatogenesis is a complicated process in which round diploid spermatogonia transforms into differentiated haploid spermatozoa with a compact nucleus, acrosome, and tail during physiological, biochemical, and morphological changes [68,69].

In our study, we found that *Cynara scolymus* L extract and *Bifidobacterium longum* improved sperm motility and viability, as well as testicular tissue growth, by increasing the number of Leydig cells, spermatids, and spermatozoa diameter. These findings support the notion that probiotics and the gut microbiota may play important roles in infertility and that *Cynara scolymus* L extract can help with infertility. Failure in chromatin packaging can expose sperm to damaging agents such as endonucleases and free radicals, ultimately contributing to DNA and telomere breakage. Telomere length is directly related to DNA damage and protamine insufficiency, which impair sperm fertility [70]. Elias found that a lack of protamine lowers disulfide bonds, which may result in a lack of sperm DNA protection [71]. Moghadam M et al. investigated the impact of diabetes on pituitary-gonadal hormonal foundation and the degree of protamine gene expression in diabetic rats. They discovered that the diabetic group's average body weight, testicle weight, testosterone hormone concentration, and protamine gene expression levels decreased dramatically compared to the control group [72].

Our findings are consistent with these findings in the diabetic rat group. Body weight, testis weight, and protamine expression levels improved significantly in the group treated with a mixture of *Cynara scolymus* L and *Bifidobacterium longum,* strengthening the possibility of co-administration of these supplements as a complementary or even alternative therapy for diabetic complications and infertility problems caused by diabetes. However, in diabetic male rats, separate administration of *Bifidobacterium longum* and *Cynara scolymus* L did not significantly increase protamine gene expression. Ghazaleh Mohammadi et al. studied the effect of a Lactobacillus helveticus and *Bifidobacterium longum* mixture on apoptosis-related genes BCL2, BAX, and caspase 3 in rats with lipopolysaccharide-induced hippocampus apoptosis. LPS dramatically decreased BCL2 gene expression while increasing Bax, the Bax/Bcl-2 ratio, and cleaved caspase-3 expression, according to their findings. They discovered that a probiotic combination (L. helveticus R0052 + B. longum R0175) reduced LPS-induced hippocampal apoptosis in rats via the gut-brain axis [73]. Artichoke extract directly suppresses inflammation and apoptosis in hepatocytes during progression of Non-Alcoholic Fatty Liver Disease (NAFLD), according to Minhee Lee et al. [74].

In the testes of diabetic rats revealed a significant decrease in *BCL2* expression and an increase in *caspase-9* expression. Administration of *Cynara scolymus* L and *Bifidobacterium longum* significantly elevated *BCL2* and modified *caspase-9* expression. Special observation techniques, such as deoxynucleotidyl transferase dUTP nick-end labeling (TUNEL) and BAX/Bcl-2 immunoblotting, have been used to demonstrate the presence of apoptosis in testis tissue. This could be a limitation that should be examined in future research.

In our study, the measured sex hormones revealed that diabetic rats had lower levels of LH, FSH, and testosterone, which were restored in our therapy groups. However, future research should be conducted to establish the expression of specific genes involved in the production of these hormones to assess the molecular signaling pathways involved in the action of *Cynara scolymus* L and *Bifidobacterium longum* therapy.

The current work is limited by the lack of a complete phytochemical investigation of *Cynara scolymus* hydroalcoholic extract. While the primary goal of the study was to investigate the therapeutic effects of the whole crude extract on male infertility, the specific bioactive components responsible for these effects were not measured. Future investigations should use techniques like High-Performance Liquid Chromatography (HPLC) or Liquid Chromatography-Mass Spectrometry (LC-MS) to perform fingerprinting or chromatographic analysis on the extract. This will provide a better knowledge of the active chemicals, such as chlorogenic acid and cynarin, and their roles in the reported biological effects. Addressing this shortcoming will improve the consistency and accuracy of the therapeutic findings linked with Cynara scolymus.

## 6 Conclusion

The study's findings showed that *Bifidobacterium longum* and *Cynara scolymus* L extracts may increase TAC levels and lower MDA levels, respectively. Moreover, our treatment increased the mRNA expression levels of *BCL-2*, *Caspase-9*, and prm1 (protamin) in the testicular tissues of diabetic male rats, which resulted in an increase in the levels of sex hormones and better cell function. Supplementation with *Cynara scolymus* L extract and *Bifidobacterium longum* improved sperm motility, survival, and aberrant sperm morphology. In summary, our research shows that *Cynara scolymus* L extract and *Bifidobacterium longum* supplement have significant therapeutic potential as antioxidants, anti-apoptotic and anti-diabetic properties, fertility index, improvement agents, and sex hormone modulators.

## Participation permission and Ethics approval

Institutional Animal Ethics Committee in Shiraz University of Medical Sciences (Shiraz, Iran) gave its approval to the study's protocols in accordance with NIH standards for treatment and use of animals (NIH publication No. 85–23, revised in 1996).

## Consent to publish

N/A.

## Availability of information and resources

This article contains all data created and examined throughout this investigation. The corresponding author will provide datasets used or analyzed during the current work upon reasonable request.

## Funding

Public, private nor nonprofit funding organizations did not provide any specific grants for this research.

## Authors’ contributions

All experiments, statistical analysis, and figure preparation were conducted by ZA, FK, ZH. The initial draft of manuscript was written by MhM, F.R, Z.E. All tests were set up and second draft of manuscript was written by SD, PM, MS, ZZ. A final manuscript proof was completed by SD who also contributed more funds to the project.

## Declaration of competing interest

The authors declare that they have no known competing financial interests or personal relationships that could have appeared to influence the work reported in this paper.

## Data Availability

The data that has been used is confidential.

## Abbreviation:

(B. Longum): *Bifidobacterium longum*
(B2m): Beta-2-Microglobulin
(FSH): Follicle-stimulating hormone
(GAPDH): Glyceraldehyde-3-phosphate dehydrogenase
(IP): Intraperitoneal
(LH): Luteinizing hormone
(Ph): Power of hydrogen
(ROS): Reactive oxygen species
(STZ): Streptozocin
(TAC): Total Anti-Oxidant Capacity
